# An improved biolistic delivery and analysis method for evaluation of DNA and CRISPR-Cas delivery efficacy in plant tissue

**DOI:** 10.1038/s41598-021-86549-9

**Published:** 2021-04-08

**Authors:** Kyle Miller, Alan L. Eggenberger, Keunsub Lee, Fei Liu, Minjeong Kang, Madison Drent, Andrew Ruba, Tyler Kirscht, Kan Wang, Shan Jiang

**Affiliations:** 1 Department of Materials Science and Engineering, Iowa State University, Ames, IA USA; 2 Crop Bioengineering Center, Iowa State University, Ames, IA USA; 3 Department of Agronomy, Iowa State University, Ames, IA USA; 4 Interdepartmental Plant Biology Major, Iowa State University, Ames, IA USA

**Keywords:** Biological techniques, Plant sciences, Gene expression

## Abstract

Biolistic delivery is widely used for genetic transformation but inconsistency between bombardment samples for transient gene expression analysis often hinders quantitative analyses. We developed a methodology to improve the consistency of biolistic delivery results by using a double-barrel device and a cell counting software. The double-barrel device enables a strategy of incorporating an internal control into each sample, which significantly decreases variance of the results. The cell counting software further reduces errors and increases throughput. The utility of this new platform is demonstrated by optimizing conditions for delivering DNA using the commercial transfection reagent *Trans*IT-2020. In addition, the same approach is applied to test the efficacy of multiple gRNAs for CRISPR-Cas9-mediated gene editing. The novel combination of the bombardment device and analysis method allows simultaneous comparison and optimization of parameters in the biolistic delivery. The platform developed here can be broadly applied to any target samples using biolistics, including animal cells and tissues.

## Introduction

CRISPR-Cas (clustered regularly interspaced short palindromic repeat—CRISPR-associated) has emerged as one of the most powerful and popular gene editing tools for both animal and plant sciences. The technology has huge potential to study gene function and speed up trait development. One method to deliver CRISPR reagents into plant cells is using DNA molecules encoding Cas proteins with conventional plant genetic transformation methods such as biolistics^1–3^. Biolistic delivery, also known as gene gun or particle bombardment, has been widely used to introduce DNA into plant tissues^4,5^, both for transient expression and stable transformation^6–8^. In addition to its use in plants, researchers have also shown biolistic delivery to be a viable strategy for delivering DNA and chemical payloads into animal cells, often penetrating skin barriers^9–11^. Both commercial and homemade gene gun devices have been utilized to bombard DNA into plant tissues^12–14^. However, to further develop effective CRISPR reagents that assist the delivery, it is important that the various components, such as different gRNAs and Cas9 expression cassettes, are evaluated quantitatively prior to being introduced into plant tissue for stable transformation.

While the gene gun is an effective device for introducing molecules into plants, the consistency and reproducibility can vary drastically from sample to sample. A number of variables have been shown to affect the efficiency of the delivery process^7,15,16^, including the condition and quality of the tissue, settings of the gene gun, the amounts of DNA and gold used, and the method for precipitating DNA onto the gold. It is almost impossible to keep the condition and quality of the tissue exactly the same, as there can be variation even within the same leaf^17^. The inconsistency and difficulty in reproducing the results on different samples pose a great challenge in quantitative comparison. To reduce the variations caused by the different tissues and improve the measurement consistency, we employed a 3D printed double-barrel (DB) device that allows two sets of test reagents to be simultaneously bombarded in parallel into the same plant tissue. The DB device was originally developed to improve reproducibility in studies of induction and suppression of cell death responses in soybeans^17,18^. In this study, the DB device allows introduction of an internal control for each bombardment, which can be used to normalize shot-to-shot variation.

Another impediment to efficient optimization and quantification of biolistic delivery is counting cells transfected with the marker gene, such as the green fluorescent protein (GFP) gene used in this work. It is important to quantify the number of fluorescent cells accurately to achieve high-quality data. Manual cell counting is often performed, but it is time-consuming and subject to variability between repeated counts or between observers. A conventional ImageJ plugin does not work well to identify the plant cells due to their unique shape and strong background autofluorescence^19^. To address these issues, we adapted the open-source software platform CellProfiler^20^ to efficiently count transfected cells, which allowed us to dramatically increase the consistency and throughput. Cell shape and morphology are diverse within and between plant species, and, compared to animal cells, we must account for significant auto-fluorescence from the cell wall and chloroplast^21^. Therefore, a workflow has been developed with many parameters optimized specifically to identify transfected plant cells. The software successfully differentiated GFP-expressing cells in both onion and *Nicotiana benthamiana* cells from the auto-fluorescent background, and the cell counting matched well with the manual counting results.

In summary, we developed a system using the double-barrel device for particle bombardment and customized CellProfiler software for cell counting. This system is shown to be used effectively for the optimization of DNA transfection conditions as well as the evaluation of efficacies of CRISPR-Cas reagents.

## Results and discussion

### Optimization of biolistic delivery with a double-barrel device

**Figure 1:**
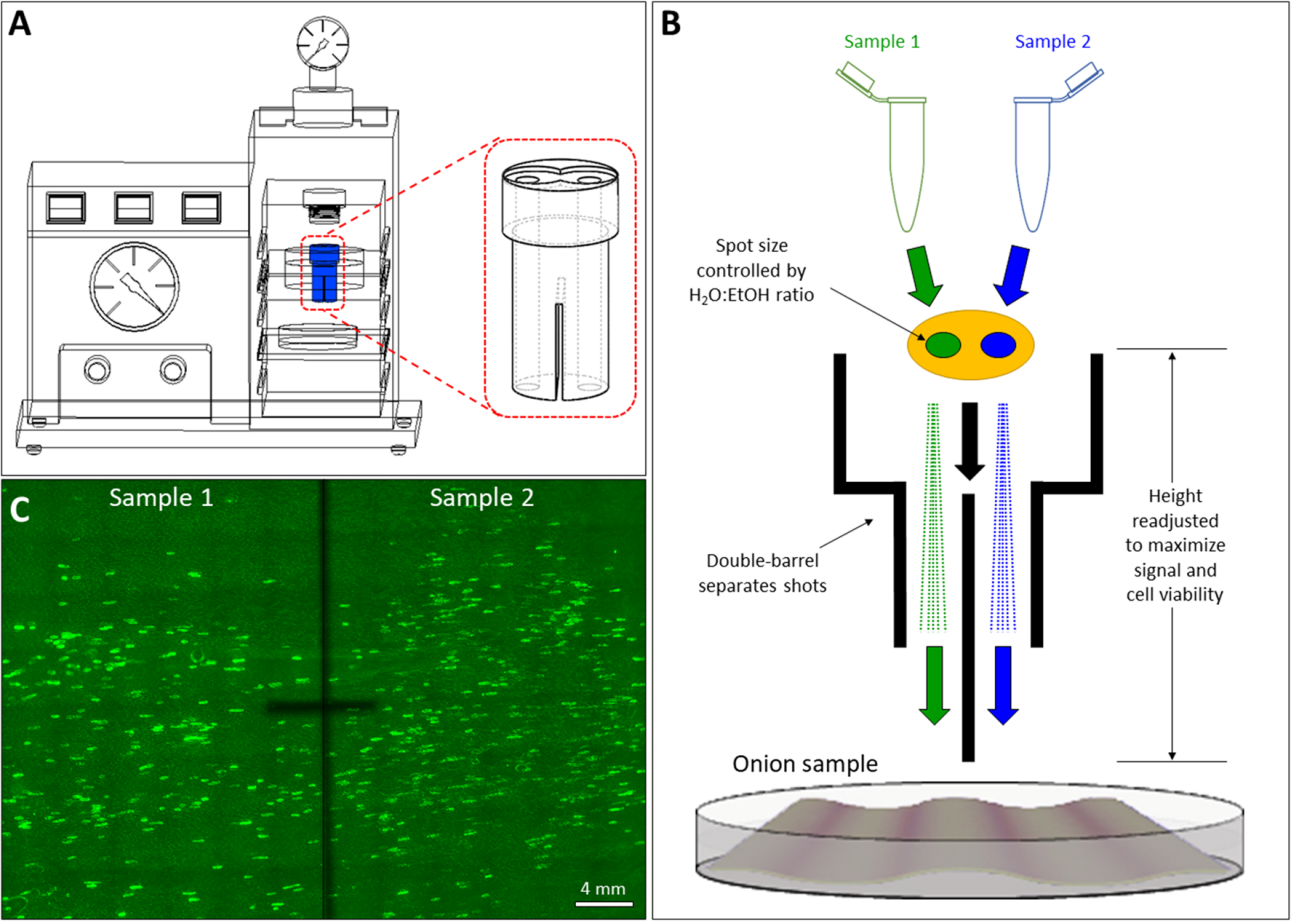
Overview of biolistic bombardment of onion epidermis tissue using the double-barrel device. (**A**) A detailed view of the double-barrel in the PDS-1000 gene gun; (**B**) schematic diagram demonstrating the modifications to the biolistic procedure to accommodate the double-barrel device; (**C**) a typical tile-scan fluorescent image of onion tissue bombarded with plasmid DNA with a gene expressing GFP. The dark cross in the middle is marked to separate the sample areas impacted by the two sides of the double-barrel device.

The double-barrel (DB) device^17^ used in this work is illustrated in Fig. 1A,B. The commercially available BioRad PDS-1000/He gene gun uses a single-barrel (SB) component that allows high velocity helium gas to propel microcarriers into the target tissues. This is effective but it can be challenging to compare the effects of different treatments because of the high variability between bombarded biological samples. We chose to adopt a custom-made DB device that has been used previously for assessing plant pathogen effectors^17^. The DB can be 3D printed so that alterations are readily implemented. The STL file for direct 3D printing is attached as Supplemental File S1, with permission from Dr. Brett M. Tyler of Oregon State University.

Our initial steps were to modify the gold/DNA loading procedure and optimize the bombardment parameters for DNA delivery into onion epidermis tissue. In a typical protocol, 100% ethanol is used to resuspend the gold/DNA before the mixture is loaded onto the center of a macrocarrier for bombardment. When 100% ethanol was used to load two gold/DNA samples with less volume onto the two half-sides of a macrocarrier, excessive spreading of the gold/DNA suspension occurred, causing misalignment of the samples with the barrels in the DB device. We solved this problem by resuspending the gold/DNA complex in 50% ethanol instead. This modification reduces the suspension droplet size via increased surface tension and allows two gold/DNA samples to load in a controlled manner on the desired positions of the macrocarrier. To ensure sample loading uniformity and reduce the prevalence of a “coffee-ring effect” from drying the ethanol–water mixture, the macrocarriers loaded with gold/DNA samples were dried under vacuum in the gene gun chamber instead of air-drying in the laminar flow hood.

We optimized bombardment parameters for the DB device using a plasmid DNA pLMNC95^22^ that carries a constitutive *gfp* gene expression cassette (Supplementary Table S1). We noted that simply copying the parameters for the SB device resulted in excessive cell death in the bombarded onion tissues. To address this, we tested several parameters including rupture disks that break under different pressures, the distance between the stopping screen and the target plate (S–T distance), and the gold quantity per shot. In a typical protocol for the SB device with onion tissue, the S–T distance is 6 cm with a 1100 lb per square inch (psi) rupture disc and 250 µg gold/shot^23^. In determining the optimal S–T distance for the DB bombardment, we chose to use a rupture disc of 650 psi and a gold quantity of 18 µg/shot after a preliminary evaluation. The effects of the S–T distance on DNA delivery were assessed by counting GFP-expressing cells 1 day after bombardment.

The cell viability was assessed by tissue staining with fluorescein diacetate (FDA). FDA is a cell-permeant esterase substrate that is often used for detecting cell membrane integrity^24^. Among the three S–T distances (6, 9, and 12 cm) tested, DNA delivery was poorest when the 6 cm S–T distance was used (Supplementary Figs. S1), which correlated to a large area of cell death in the 6 cm sample as determined by FDA staining (Fig. S1B). Because dead cells would not express the *gfp* gene, the low fluorescent cell counts in the 6 cm sample were the consequence of the large number of dead cells. The amount of gold particles per shot also affected the DNA delivery and cell viability, but to a lesser degree (Fig. S2). More gold particles could deliver more DNA molecules but could also cause more cell damage. Our side-by-side comparison of different gold particle amounts per shot when using a 650 psi rupture disk and 12 cm S–T distance suggested that 18 µg/shot gold appeared to provide consistent results in DNA delivery with the least cell damage (Fig. S1E,F).

**Figure 2:**
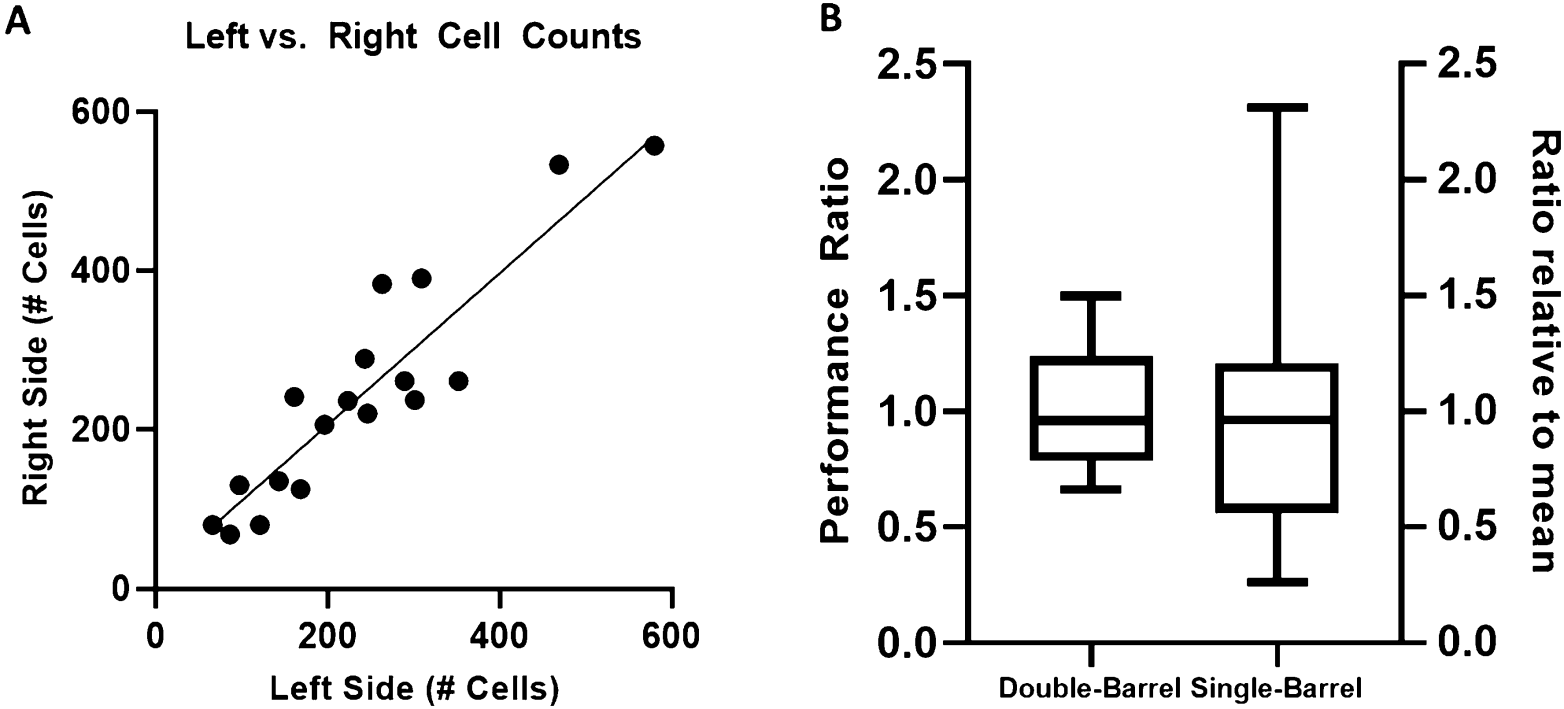
Comparison of GFP-expressing cell numbers from onion tissue bombarded with a single- or double-barreled biolistic device. (**A**) Scatter plot comparing the number of fluorescent cells on the left and right side of a sample when the treatment is identical. The cell counts from both sides had a linear correlation with a slope of 0.95 and coefficient of determination (*R*^2^) of 0.79; (**B**) box plots demonstrating the effect of the double-barrel on controlling for sample variation. The left box plot shows the performance ratio (experimental side/control side) of the same samples from (**A**) (n = 21) after applying the internal control to each sample. The right box plot shows the same data as if they were single-barreled shots, with the number of cells shown as a ratio to the mean (251 cells) for comparison. Box plots show the full range and the inner quartiles. Standard deviations are 0.26 for double-barrel, and 0.52 for single-barrel.

To avoid any residual gold/DNA cross contamination, we included an additional step of barrel rinsing between each bombardment of the samples. Fig. S3A shows cross contamination when the barrel was not cleaned, while Fig. S3B shows the much-reduced incidental fluorescence when the barrel was rinsed. To further measure the consistency of the DB device, we bombarded 24 onion samples over the course of several days using identical DNA, reagents, and bombardment parameters. The number of GFP-expressing fluorescent cells on the left and right sides of the bombarded onion samples were counted and compared. Fluorescent cell counts from both sides had a linear correlation with a slope of 0.95 and coefficient of determination (*R*^2^) of 0.79 (Fig. 2A). This plot is similar to other reports in the literature on the range of variance when using the DB device to deliver identical reagents side-by-side, confirming the reliability and consistency of this procedure^18,25,26^. Figure 2B shows the reduction in variability when using the DB device. To calculate the effectiveness of the delivery without using the DB device, one would count the number of transfected cells and then define the effectiveness of the procedure by the average and standard deviation (SD) of the set. However, with the DB device we can use the ratio between the number of cells on each side of a sample instead, representing how many cells were transfected on the experimental side (right side) in comparison to the control side (left side). We have defined this as the “performance ratio” of that sample. Figure 2B shows the effect of using the DB performance ratio vs. a conventional SB method. For comparison, the single-barrel statistics are represented by their ratio to the mean of the set (251 cells) rather than as raw numbers. The box and whisker plot shows the entire range as well as the inner quartiles. By applying the DB system, the standard deviation was reduced by half (0.52 for normalized cell counts; 0.26 for the DB performance ratio).

These results show that the DB device can be used to dramatically reduce errors from variance in the target tissue. While the sample can change its quality from a variety of conditions, the two sides of the same sample were strongly correlated. The reduction in variation when applying this internal control allows for new experimental designs that take advantage of the lower minimum sample count required to reach statistical significance.

### Customizing CellProfiler for enabling intricate cell counting

After demonstrating the ability of the DB device to reduce error, we wanted to further improve the speed and consistency of cell counting to enable higher throughput testing. Manual counting took several hours per batch of samples and varied from person to person, adding additional sources of error to the data. Standard software packages like ImageJ are available, but plant cells such as the onion epidermis have unique profiles that make them more difficult to count^19^. To address this challenge, we chose the open-source CellProfiler^20^ after surveying several software options. CellProfiler is a modular program with a user-friendly interface, allowing us to more easily construct a method to track and count the cells automatically. The program offered various pre-made modules to process images. However, customization was needed to analyze GFP-expressing onion epidermis cells at the level of brightness obtained from our microscope.

While the full software pipeline is described in the supporting information, the process of optimization is generalizable. To start, basic settings in the “Identify Primary Objects” module were established based on the size and brightness levels of the cells. To help reduce the effect of a noisy, heterogeneous background, we then applied a Gaussian blur filter to the images, which averages local brightness levels, effectively suppressing small brightness peaks. This effect is shown in Fig. S4. The next step was to establish a cut-off threshold for distinguishing a transfected cell from the background. Because brightness levels of the background changed from image to image, we applied an algorithm to minimize cross entropy by automatically calculating a threshold for each image. This was then empirically adjusted using a multiplicative threshold correction factor. Finally, in the continuous layer of onion cells, fluorescent cells often cluster together. The important “de-clump” function in CellProfiler has the capability to separate these cells. The parameters for this module were determined empirically, and a sample of this is shown in Fig. S5. These same steps are broadly applicable to other plant systems of different cell shapes.

**Figure 3:**
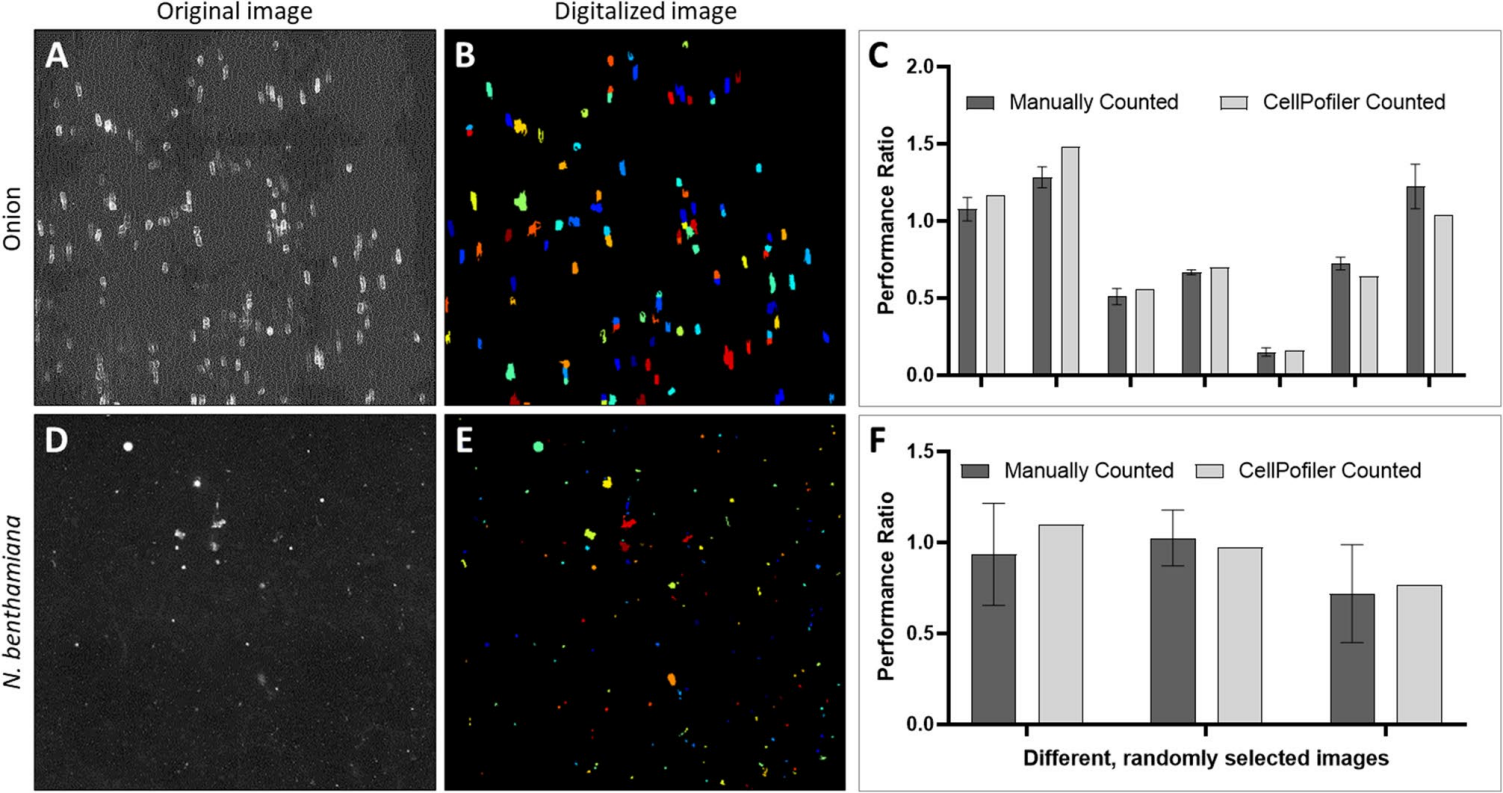
Algorithm validation. (**A**) The original image of the GFP fluorescent onion cells, shown in black and white to reflect the greyscale nature of the CellProfiler algorithm; (**B**) an image produced by the CellProfiler counting, showing which cells were counted by the software. Different colors indicate distinct cells; (**C**) statistics comparing the performance ratio of several randomly selected images counted by hand as well as by the software. Note that the CellProfiler counts have no error bar as the software is self-consistent, which is in contrast to the manual counting; (**D**–**F**) the protocol for counting cells was optimized and repeated for *N. benthamiana* cells, which includes both epidermis and trichome cells.

When processing large numbers of images, it is inevitable that some images will fall outside the bounds of the software and be miscounted. In addition to the number of cells, the software also outputs many other points of data that can be used to quickly assess the counting quality. The most obvious is exporting an image overlaying the counted areas with the original image and visually checking them, as shown in Fig. 3 and Supplemental Figs. S4–S5. Another point of quality control is to check the significant deviations in some of the algorithmically generated values. For example, if the threshold chosen for a particular image deviated from the total average threshold, it was flagged for a closer visual inspection.

To ensure the accuracy of the program, we selected a subset of images to validate manually and compared the manual performance ratio to the software’s performance ratio (Fig. 3A–C). While the software was able to consistently calculate a value within the range of the manually counted values, the program was also able to identify roughly 20% more cells than the manual count (Supplemental Table S2). We believe there are two likely reasons for this. The first is that we calibrated the software to count slightly dimmer cells to compensate for very bright images, and, on average, this led to a higher count compared to the manual count because it is more consistent in counting cells above a certain brightness level. The second is that the de-clumping is splitting cells determined to be too long, when the aspect ratio of these onion epidermis cells is relatively high. However, because these adjustments affect both sides of the plant sample data equally, they have a negligible effect on the final performance ratio. It should also be noted that while the manual counts in Fig. 3C have error bars due to the variance from multiple people counting, the considerably faster algorithm counts were consistent and thus did not have error bars. The software also allows us to capture the brightness (integrated density) of cells on each side rather than just the number of cells, which should be a more accurate indication of GFP expression. However, brightness values are much more difficult to verify when calibrating the software and are strongly correlated with the number ratio regardless (Fig. S6), therefore we continued to use the ratio between the number of cells counted.

The broader applicability of the software was tested by applying it to the model plant *N. benthamiana.* The procedure was slightly altered for the smaller epidermal pavement and trichome cells in *N. benthamiana* by iterating on parameters such as cell size and threshold and adding an additional module to enhance the small fluorescent features. As with the onion cells, these were verified by manual counting. Figure 3D–F shows that the performance ratio was accurately captured by the software as well. Due to the small cell size and uniformity of the software counting, the raw number of cells counted was markedly higher than the manual counting, although the performance ratio is within the range counted (Fig. 3F).

### Application of the double-barrel CellProfiler system

**Figure 4:**
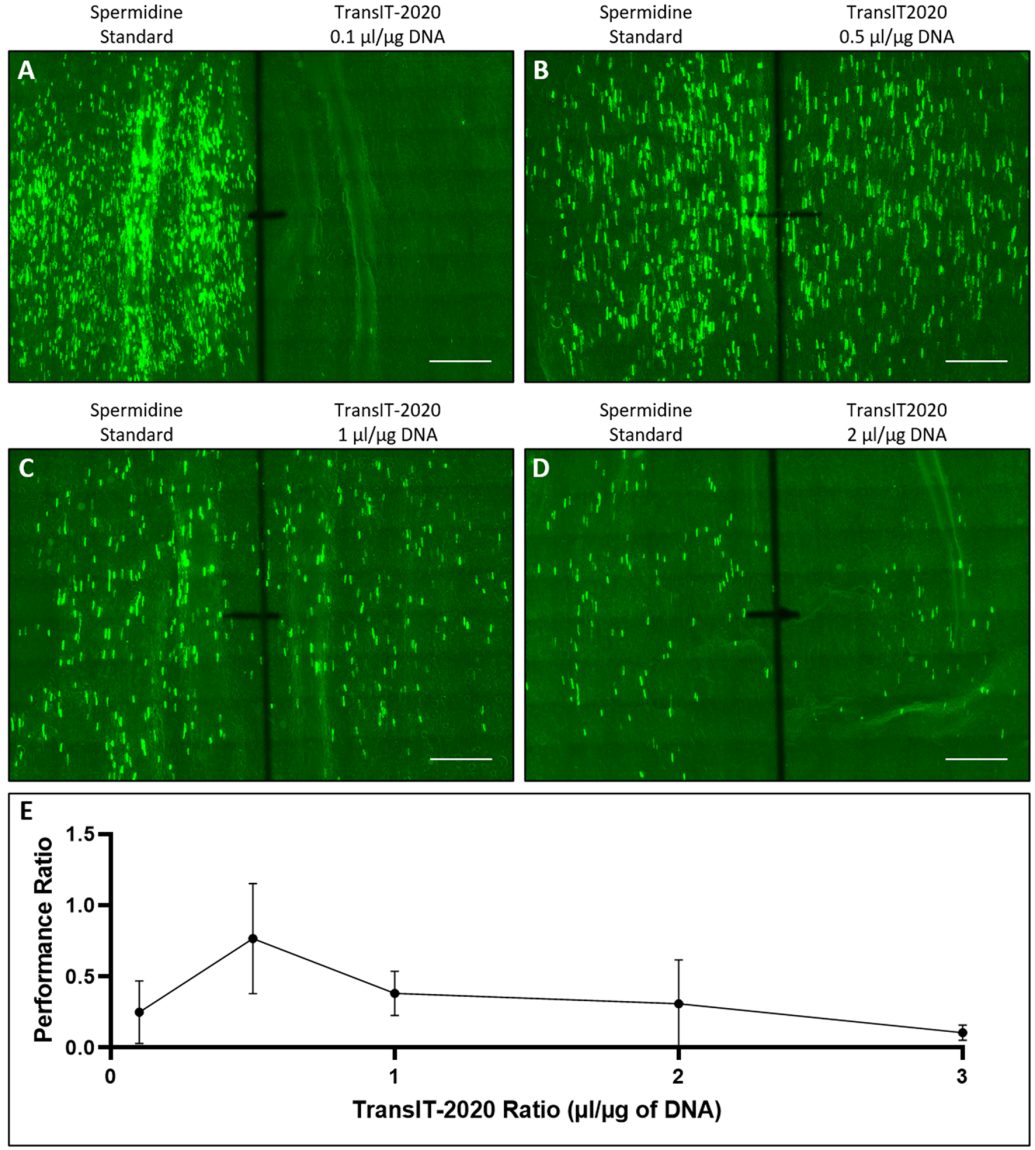
Effect of *Trans*IT-2020 quantity on DNA delivery. (**A**–**D**) DNA delivery measured by GFP expressing cells using different quantities of *Trans*IT-2020 (µL/µg DNA), with spermidine as delivery control. (**E**) Performance ratios for different amounts of *Trans*IT-2020 showing optimized ratio of 0.5 µL/µg. Scale bar is 4 mm.

We first applied the double-barrel/CellProfiler (DB-CP) system to evaluate the effectiveness of transfection reagent *Trans*IT-2020 (Mirus Bio LLC, Madison, WI, USA) for DNA delivery into plant cells. The majority of bombardment procedures for plants reported in the literature used spermidine as the standard reagent to precipitate DNA onto gold particles^5,23,27^. In recent years, however, *Trans*IT-2020 has emerged as an alternative reagent for introducing DNA^28^ as well as ribonucleoprotein^29–31^ into plant tissues. Interestingly, despite its successful application in plants, there has been scant information for optimizing the coating of DNA onto gold with the *Trans*IT-2020. Using spermidine as an internal control, we compared the effect of different *Trans*IT-2020/DNA ratios when delivering the *gfp* gene construct (Fig. 4). Among four quantities (0.1, 0.5, 1 and 2 µL per µg DNA for each coating reaction), 0.5 µL *Trans*IT-2020 per µg DNA in each gold coating reaction seemed to be optimal (Fig. 4E) and compared most favorably with spermidine (Fig. 4B,E). Note that the same spermidine control in this set of samples showed markedly different DNA delivery outcomes (Fig. 4A–D left half), further demonstrating significant variation between biological samples. This highlights the importance of using the DB device and the inclusion of an internal control for each treatment evaluation. Our DB-CP system allows significant reduction of result variance and enables rapid evaluation and optimization of delivery reagents. In the *Trans*IT-2020 system, the DB-CP enables direct comparison of different coating reagents, which would be otherwise difficult in a two-plasmid reporter assay.

**Figure 5:**
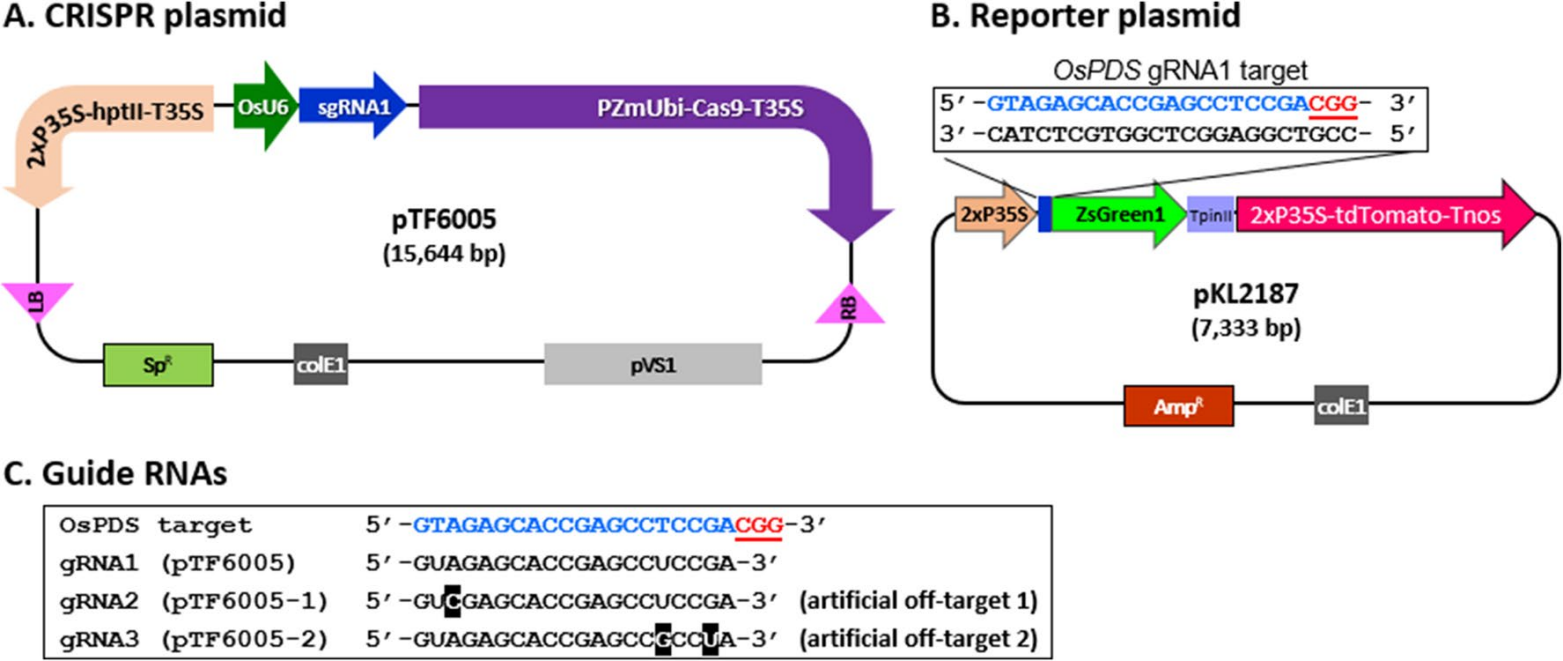
Schematic representation of two plasmids used for the evaluation of CRISPR reagents. (**A**) CRISPR plasmid pTF6005^28^ that carries a Cas9 expression cassette under the control of maize ubiquitin promoter and Cauliflower Mosaic Virus (CaMV) 35S terminator (T35S); OsPDS gRNA1 is regulated by OsU6 promoter; hygromycin resistance gene (*hpt II*) is driven by 2 × CaMV 35S promoter (P35S) and terminated by T35S. RB, T-DNA right border; LB, T-DNA left border; Sp^R^, spectinomycin resistance gene; ColE1 ori, high copy number origin of replication for *E. coli*; pVS1, origin of replication from plasmid VS1 for *Agrobacterium*. (**B**) The reporter plasmid pKL2187 has genes for the red fluorescent protein tdTomato and the green fluorescent protein ZsGreen1. Transcription of the tdTomato gene is driven by a 2X P35S and terminated by an *Agrobacterium* nopaline synthase terminator (Tnos). The encoded tdTomato protein has an SV40 nuclear localization signal at the N terminus. Transcription of the ZsGreen1 gene is driven by a 2X P35S promoter and terminated by a potato protease inhibitor II terminator (TpinII). The translation start codon is preceded by a TMV Ω translational enhancer and is immediately followed by the target sequence of the OsPDS-gRNA1 expressed from pTF6005. The open reading frame for the flexible peptide linker 2X (GGGGS) and ZsGreen1 is out-of-frame by 1-bp with the start codon and is not translated; however, indel mutations at the gRNA target site can bring the ZsGreen1 gene in-frame and restore green fluorescence. Amp^R^, ampicillin resistance gene. The plasmid pKL2188 is identical to pKL2187 except that the ZsGreen1 gene is in-frame with the start codon. Blue letters, gRNA target sequence; underscored red letter, PAM sequence. (**C**) Three guide RNAs tested for editing efficiency. All gRNAs were driven by OsU6 promoter in the CRISPR plasmid shown in (**A**). gRNA1, on-target gRNA; gRNA2 and gRNA3, artificial off-target gRNAs with one or two mismatches (white letters in black boxes).

We then took advantage of our DB device to compare the editing efficacy of three gRNAs utilizing a test system where editing restores the correct translation of an out-of-frame fluorescent protein gene^32^. The CRISPR plasmid pTF6005^28^ (Fig. 5A) has the Cas9 gene and a gRNA that matches exactly the target site in the reporter plasmid pKL2187 (Fig. 5B,C). This is hereafter referred to as gRNA1. To further validate our platform, two additional gRNA sequences, gRNA2 (pTF6005-1) and gRNA3 (pTF6005-2), were designed with mismatches in their gRNA sequences (Fig. 5C). The sequence of gRNA2 has a single mismatch distal to the PAM (protospacer adjacent motif) sequence. Therefore, gRNA2 is predicted to have a lower editing efficiency compared to gRNA1. Furthermore, gRNA3 has two mismatches proximal to the PAM sequence and is expected to have a much poorer editing efficiency compared to the other two gRNAs. The reporter plasmid pKL2187 (Fig. 5B, Fig. S7) has a start codon and OsPDS sgRNA1 target sequence upstream of a *gfp* gene (ZsGreen1) that is out-of-frame by 1 bp relative to the start codon. Because of the frameshift and premature stop codon immediately after the gRNA target site, the ZsGreen1 gene is not translated to produce functional GFP, thus no green fluorescence should be detected. When the pTF6005-series Cas9 plasmids and pKL2187 are co-bombarded into plant tissue, edited cells will show green fluorescence because the expression of GFP from some of the pKL2187 molecules is restored after editing and repair of the target site. A *gfp* gene in-frame version of pKL2187, pKL2188, was used as a positive control to demonstrate the upper limit of ZsGreen1 expression from pKL2187. Both plasmids, pKL2187 (Fig. 5B) and pKL2188, have a constitutively expressed red fluorescent protein (tdTomato) gene, which was used to identify transfected cells and served as an internal control for the GFP expression normalization.

**Figure 6:**
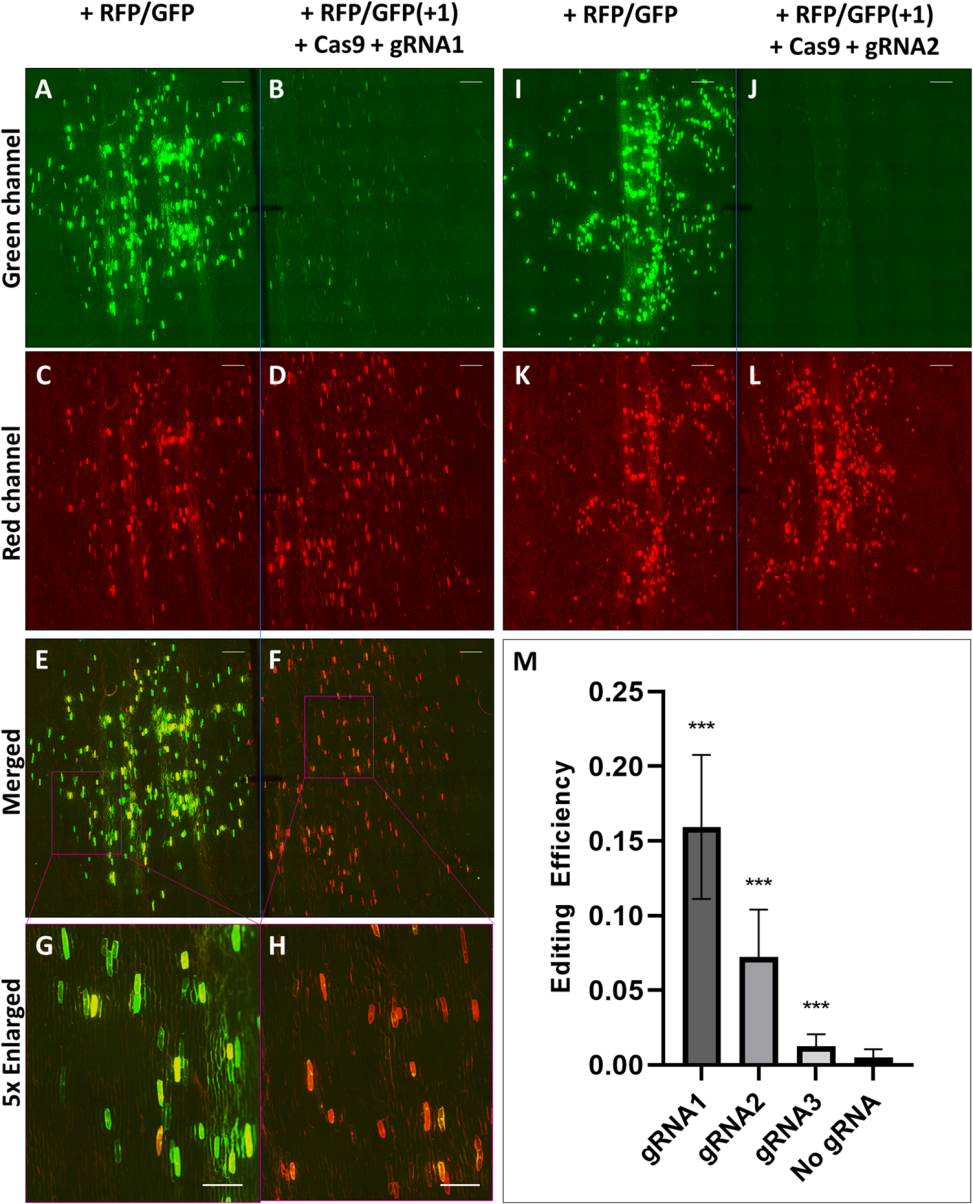
CRISPR-Cas9-mediated editing of a reporter plasmid. The reporter plasmid pKL2187 [(+ RFP/GFP(+ 1)] constitutively expresses RFP but expresses GFP only if editing of the GFP leader sequence restores the correct reading frame, whereas the control plasmid pKL2188 (+ RFP/GFP) is almost identical to pKL2187 except that GFP is expressed without editing. A representative onion sample bombarded on the left side (**A**,**C**,**E**,**G**,**I**,**K**) with plasmid pKL2188 as a positive control, and on the right side with the mixture of reporter plasmid pKL2187 and a CRISPR plasmid carrying either perfect match gRNA1 (**B**,**D**,**F**,**H**) or one nucleotide mismatch gRNA2 (**J**,**L**); (**M**) graph showing editing efficacy on onion samples for all gRNAs tested and a negative control, all relative to the pKL2188 positive control. Scale bar for (**A**–**F**,**I–L**) is 4 mm, (**G**,**H)** is 2 mm.

The results of using the DB-CP system to evaluate the CRISPR reagents for gene editing efficacy are shown in Fig. 6 and Fig. S8. A constant in-frame positive control was used as a standard to compare to the efficacy of different gRNAs and show the upper limit of fluorescent protein expression (Fig. 6A,C,E,G,I,K). The other half of the tissue was co-bombarded with an out-of-frame *gfp* plasmid (pKL2187) and the Cas9 construct with the relevant gRNA sequence. Figure 6B,D,F,H show the editing effects of gRNA1, which contains no intentional errors, and Fig. 6J,L show that of a single mismatch gRNA2. Additional images in Fig. S8 show the effect of double mismatch gRNA3 (Fig. S8B,D) and the negative control (Fig. S8F,H) in which the reporter gene was bombarded, but the Cas9 construct was produced with off-target gRNA (A845B^33^) in order to show incidental fluorescence, if any.

The relative editing efficacies of gRNA1, gRNA2, and gRNA3 was determined by comparing the numbers of green fluorescent cells generated by the editing system to the number of green fluorescent cells in the positive controls, normalized to the number of red fluorescent cells that represented the total number of transfection events. This was done by first counting the numbers of green and red cell in each bombarded sample on each side. The number of green cells were then divided by the number of red cells to determine the ratio of *gfp*-expressing cells out of the total transfected cells. Subsequently, the green/red ratio of a particular treatment was divided by the green/red ratio of the in-frame *gfp* gene construct pKL2188 (the positive control, Fig. 6A,C,E,G,I,K) to calculate the editing efficacy.

As shown by Fig. 6M, the three tested gRNAs showed distinct performances when compared to the in-frame positive control (pKL2188) after only a small number of replicates (10 in each case). The relative performance, termed the editing efficacy, for the co-bombardment of the reporter plasmid pKL2187 and the CRISPR reagent with each gRNA is 0.16 ± 0.05 (mean ± SD) for gRNA1, 0.07 ± 0.03 for gRNA2, and 0.01 ± 0.01 for gRNA3. The performances of all gRNA sequences were statistically significantly different from each other (*P* < 0.001, t test). The negative control, comparing the in-frame positive control to a sample containing only the reporter plasmid (pKL2187) and an off-target gRNA, shows virtually no green fluorescence.

These results demonstrate that the DB-CP system can be used to reliably compare two treatments simultaneously in a biolistic experiment. It can be used to compare different chemicals for coating DNA onto gold (such as spermidine vs TransIT-2020) and evaluate various components needed for an effective CRISPR reagent prior to plant transformation.

## Conclusion

Here we report an improved biolistic delivery and analysis method with the DB device and a customized cell counting software. This system reduces the data variance caused by biological sample variations and image analysis discrepancies, thus reducing the number of replicates required to obtain meaningful statistics. While the DB device has been used previously to study genes involved in inducing or suppressing cell death in plants^17,18,34^, to our knowledge it has not been adapted to optimize the biolistic delivery process itself. When accompanied by an accurate and reliable counting software, the approach becomes a powerful tool for assessing different transfection protocols. We have successfully demonstrated the application of DB-CP for the evaluation of chemical *Trans*IT-2020 and CRISPR-Cas reagents, showing that conditions and parameters can be efficiently and accurately assessed with a relatively small number of samples. This procedure opens up possibilities for many parameters to be tested and improved, such as the binding agent, the particles used, and impact from other variations in the preparation protocols.

## Materials and methods

### Single barrel bombardment

The standard single barrel for the BioRad PDS-1000/He (Bio-Rad Life Science, Hercules, CA, USA) was assembled as described in the Bio-Rad manual, available from the company website, with the stopping screen holder placed between the spacer rings. Gold particles (0.6 µm, Bio-Rad Life Science) were washed with isopropanol as described by Sawant et al.^35^ followed by washes with water and final resuspension in water to give the indicated concentration. Gold aliquots were transferred to 1.5 mL microfuge tubes and stored frozen until use. To precipitate DNA onto gold, 50 µL of sonicated 15 mg/mL gold was combined with 0.5 µg plasmid DNA pLMNC95, encoding a gene for an endoplasmic reticulum-localized GFP (ER-GFP)^22^. This was followed by the addition, while vortexing, of 50 µL 2.5 M CaCl_2_ and 20 µL 0.1 M spermidine (Thermo Fisher Scientific, Waltham, MA, USA). Vortexing was then continued for an additional 1 min. The gold/DNA complex was collected with a brief centrifugation, washed with 70% ethanol, and resuspended in 2× the original volume of 100% ethanol. Tubes of the DNA/gold precipitate were briefly sonicated to achieve a uniform suspension and then 10 µL aliquots were removed during vortexing and spread on the center 1 cm of macrocarriers (Bio-Rad Life Science or Analytical Scientific Instruments, Richmond, CA, USA), which were then allowed to dry in the laminar flow hood.

White onions (*Allium cepa*) were obtained from a local grocery store. Sections of epidermis ≥ 2 × 2 cm were removed from the inner surface of onion scales immediately before shooting and placed on droplets of 0.5 mM 2-(*N*-morpholino) ethanesulfonic acid (MES) buffer pH 5.6 (50 µL total volume) on the surface of agar plates containing 0.7% Difco Bacto Agar in 0.5 mM MES pH 5.6. Transfection was performed using 650 psi rupture disks (Bio-Rad Life Science or Analytical Scientific Instruments) and a 6 cm distance to target. Plates were wrapped with parafilm after shooting and incubated in the dark for approximately 24 h before observation.

### Double-barrel device

The double-barrel device^17,18^ for the PDS-1000 particle gun was kindly obtained from Dr. Brett M. Tyler of Oregon State University (https://bpp.oregonstate.edu/users/brett-tyler). With permission of Dr. Tyler, we recreated a 3D model of the device and generated a STL file suitable for 3D printing (Supplementary File S1). Its actual appearance is shown in Supplemental Fig. S9.

For the DB device, several adjustments were made to the procedure, most significantly the smaller aliquot on the macrocarrier (leading to reduced material per shot) and the S–T distance. Gold aliquots were briefly sonicated and 20 µL of 0.1 µg/µL pLMNC95 was added per 50 µL gold (12 mg/mL gold solution). For precipitation with CaCl_2_ and spermidine, 50 µL 2.5 M CaCl_2_ and 20 µL 0.1 M spermidine were added to the gold/DNA mixture as it was being vortexed. After continued vortexing for 1 min the tubes were centrifuged to pellet the gold/DNA complex, and the pellets were partially resuspended in 70% ethanol. The supernatant was removed, and the gold particles were finally resuspended in twice the original volume of 50% ethanol/water mixture. For precipitation of DNA onto gold with *Trans*IT-2020 transfection reagent (Mirus Bio, Madison, WI, USA), the indicated amount of *Trans*IT-2020 was added to the gold/DNA mixture during vortexing and vortexing was continued for 10 min at room temperature. The tube was then centrifuged briefly to collect the gold/DNA complexes, which were then resuspended in twice the original volume of water.

Aliquots (3 µL) of the gold/DNA suspension were removed during vortexing and quickly transferred to positions on the macrocarriers that had previously been marked so that they would line up with the barrels of the double-barrel device. Macrocarriers were transferred to the gun chamber within a minute of depositing the gold/DNA suspensions and maximum vacuum (930 mbar) was applied for four minutes to dry. The rapid drying is critical when using ethanol–water mixtures to avoid the “coffee-ring” effect.

The double-barrel device replaced the standard adjustable stopping screen support and spacer rings in the stainless-steel fixed nest (Fig. 1A), as described by Kale and Tyler^36^. This assembly was then placed in the brass adjustable nest. To avoid overlap of shot patterns, a divider of the appropriate length was placed in the slot at the bottom end of the DB device, such that the bottom edge of the divider was just above the agar plate. The petri dish was marked with a cross hatch that was aligned with the divider so that the imaging would be centered on the same location later as shown in Fig. 1C. The macrocarriers were placed on the double-barrel device with the gold/DNA spots over the barrel openings. Tissue samples were shot at the 12 cm distance (the longest available) unless otherwise noted, using 650 psi rupture disks (Bio-Rad or Analytical Scientific Instruments) and 930 mbar vacuum. To prevent carryover of gold/DNA complexes from previous sets of bombardments, barrels were rinsed with distilled water followed by rinsing with 70% ethanol and drying under vacuum in the gun chamber. Bombarded plates were wrapped with parafilm and incubated in the dark at room temperature for approximately 24 h before observation.

### Preparation of plant material

Sections of onion epidermis (≥ 3 × 4 cm, with the long axis perpendicular to the scale veins) were removed from the inner surface of onion scales and placed on agar plates as described above for the single barrel device. *N. benthamiana* plants were grown in soil in a growth room under a 16 h light/8 h dark cycle. Leaves (≥ 3 cm wide) of small plants were excised and placed on agar plates such that the leaf midrib was between the gold impact sites similar to as described by Kale and Tyler^17^ for soybeans.

### Cas9 editing assay

Four CRISPR-Cas9 plasmids were used for the editing assays (Table S1). The CRISPR-Cas9 construct with OsPDS gRNA1, pTF6005 (Fig. 5A) was described previously^28^ and two additional constructs, pTF6005-1 and pTF6005-2, were made by cloning gRNA2 and gRNA3 sequences, respectively (Fig. 5C), into pDW3586^28^ using *Bsa*I. These constructs carry a gRNA targeting the OsPDS gRNA1 target site in pKL2187 (Fig. 5) and are expected to result in GFP expressing cells if editing occurred. A845B^33^ was the off-target plasmid with a gRNA that did not target the reporter plasmid and thus served as a negative control for editing.

Precipitation of the Cas9 and reporter plasmids onto gold was done as above except that the total amount of DNA was increased 10.5-fold. The molar ratio of Cas9 plasmids to reporter plasmids was 2:1. Samples were incubated at 30 °C in dark for 2 days prior to observation.

### Imaging

Images were taken using a Leica DMi8 inverted microscope with an automated stage and digital camera. Low magnification was used in conjunction with the tilescan feature to merge multiple images into a mosaic. A typical image is shown in Fig. 1C. Fluorescent images were captured with a FITC filter (excitation: 460–500, emission: 512–542 nm) or a Texas Red filter (excitation 542–582, emission: 604–644 nm). For gold amount optimization, fluorescein diacetate staining was used to identify areas of plant tissue that had been killed by the gold impact^24^. The fluorescein diacetate stain (Thermo Fisher Scientific) used in these images was diluted in acetone to 5 mg/mL. 100 µL of this solution was mixed with 10 mL of water in which the onion tissue was submerged.

### Cell analysis

Cell analysis was done using CellProfiler 3.1.9 for Windows and was run on a Windows 10 PC. The modules included in the pipelines were used without customization beyond the available options included in the software. Detailed explanations of the modules used in the pipeline are described in the Supplemental Methods S2 and S3. Outside of adjustments for cell size, the only significant difference between the onion and *N. benthamiana* pipelines is the addition of the “Enhance or Suppress Features” module to enhance the small fluorescent spots for *N. benthamiana*.

### Statistical analysis

The chart generated for Fig. 6M shows two values and standard deviations calculated differently. First, the numbers of red and green cells were counted for each treatment. Then, the green/red cell ratio was calculated for each sample. For the on-target gRNA (+ gRNA), the green/red cell count ratio was divided by that of the positive control (i.e., in-frame construct). The mean and standard deviation were calculated from the samples with a sufficient number of cells (> 180 for positive control, and > 90 for the negative control). For the off-target gRNA comparison, no DB experiments were performed with the in-frame control, thus paired sample analysis was not possible. Thus, the mean and standard deviation of the green/red cell ratio were individually calculated for the off-target gRNA and the in-frame control. Finally, the editing efficacy ratio (Q) was calculated by dividing the green/red cell ratio of the off-target gRNA (a) with that of the in-frame control (b). The standard deviation of this editing efficacy was calculated via the error propagation formula for division and multiplication^37^, shown below. The variables $\delta a$ and $\delta b$ are the respective standard deviations of those sets,

$$Q=\frac{a}{b}, \frac{\delta Q}{|Q|}=\sqrt{{\left(\frac{\delta a}{a}\right)}^{2}+{\left(\frac{\delta b}{b}\right)}^{2}}$$

## Supplementary Information

Supplementary Information

Supplemental File S1
