# Supplementary material for: An improved biolistic delivery and analysis method for evaluation of DNA and CRISPR-Cas delivery efficacy in plant tissue
Source: Sci Rep. 2021 Apr 8;11:7695. doi: 10.1038/s41598-021-86549-9 (PMC8032657; doi:10.1038/s41598-021-86549-9)
Supplement: Supplementary file 1 — Supplementary Information [file 41598_2021_86549_MOESM1_ESM.pdf]

Supplementary Materials

An improved biolistic delivery and analysis method for evaluation of DNA and CRISPR-Cas delivery efficacy in plant tissue

Kyle Miller[1], Alan L. Eggenberger[1,2], Keunsub Lee[2,3], Fei Liu[1], Minjeong Kang[2,3,4], Madison Drent[1], Andrew Ruba[1], Tyler Kirscht[1], Kan Wang[2,3]*, Shan Jiang[1,2,3]*

[1]Department of Materials Science and Engineering, Iowa State University, Ames, Iowa, USA

[2]Crop Bioengineering Center, Iowa State University, Ames, Iowa, USA

[3]Department of Agronomy, Iowa state University, Ames, Iowa, USA

[4]Interdepartmental Plant Biology Major, Iowa State University, Ames, Iowa, USA

*Co-Corresponding authors:

Kan Wang, kanwang@iastate.edu

Shan Jiang, sjiang1@iastate.edu

Supplementary Materials and Methods

Methods S1

Double-barrel Optimization Details

During initial use of the double-barrel (DB) device, the bombardment caused severe damage to the onion epidermis and produced very poor fluorescence. The initial conditions used for single-barrel (SB) bombardment had a stopping-screen to tissue (S-T) distance of 6 cm, with 62.5 µg of gold/shot and an 1100 psi rupture disk. After reducing the rupture disk pressure to 650 psi, these conditions produced the results shown in Fig. S1A and B. The rest of Fig. S1 shows that by increasing the S-T distance up to 12 cm, the tissue damage was reduced while maintaining strong delivery as shown by the number of fluorescent cells. While the 9 cm S-T distance also shows slightly more transfected cells, the 12 cm resulted in more living tissue on average and was chosen as the parameter used moving forward.

The fluorescein diacetate stain used in these images was obtained from Fischer and diluted in acetone to 5 mg/ml. One hundred (100) µl of this solution was mixed with 10 mL of water in which the onion tissue was submerged[1].

The gold amount was investigated in conjunction with the S-T distance, and the results are shown in Fig. S2. These results were less definitive. While the lowest value of gold (12 µg/shot) showed fewer fluorescent cells, it was difficult to distinguish between the 18 and 22.5 µg/shot amounts. This is significantly less than the amount used in SB bombardment, but that is primarily because the aliquot is much smaller.

Methods S2

CellProfiler Pipeline Optimization

When using the CellProfiler image analysis, some parameters needed to be empirically determined to optimize the CellProfiler (CP) pipeline. A key step in the optimization of the software was applying a Gaussian blur. This is a common pre-processing step in many computer image analysis programs that reduces an image’s noise along with the level of detail in the image. This is essential to apply to images of onion epidermis, where the cell walls have significant autofluorescence and interfere with the software’s counting. Fig S4 shows that a slight blur altering the image significantly improves the accuracy of the counting. The strength of the blur is measured by the sigma value of the Gaussian function. Several sigma levels of Gaussian blur were tested, and ultimately a sigma of 4 was chosen.

The second factor tested involved the de-clumping function of the software. As part of the “Identify Primary Objects” module, the software is able to split larger areas above the brightness threshold into smaller cells. It can do this based on multiple different criteria. The first is shape-based, which looks for curvature at the border of the cluster to separate cells. The other is intensity-based, which looks for patterns in intensity within the cell cluster. Fig. S5A outlines their effect on the de-clumping process. These results confirm that the shape-based separation is

the most accurate. In onion cells, the brightness pattern is concentrated at the edges rather than the centers of the cells, which is the opposite of how animal cells would distribute the fluorescence, and what the software would assume we are working with when using the intensity-based separation. It is worth noting that the software uses the de-clumping method twice and is able to combine shape-intensity separation methods if that is desired. Based on some additional testing and the reasons specified so far, this was unnecessary as the "shape" method always should be used (this is effectively a "shape-shape" combination).

The de-clumping function also allows users to provide a minimum distance between cells to be separated. This sets how aggressively the software separates clusters into smaller cells. For example, a minimum distance of zero would put no limits on the de-clumping, and cells could be fragmented where no border actually exists. An extremely large minimum distance would effectively stop the de-clumping function entirely, as every point in the cluster would be deemed too close to split into multiple cells. The results shown in Fig. S5B demonstrate the process for determining the appropriate minimum distance. In our work, we identified this value to 15 pixels, slightly less than the center-to-center distance of two adjacent cells.

In addition, the software can quantify the brightness values contained in the cells. One could argue that this better represents the delivery of DNA to the cells than just counting the number of bright cells. However, Fig. S6 shows that the performance ratios of these two methods are strongly correlated. We selected the counting because it is nearly impossible to check the brightness manually, whereas the number of cells is more easily confirmed.

No algorithmic solution can be a perfect replacement for the human eye. The goal of the software is to analyze the image as a whole and in particular the relationship between the two sides, as accurately as possible. This means tuning the available parameters so that the images can be analyzed fairly, within the bounds of the parameters of the software. Images with different cell sizes or differently shaped cells would be far less accurately counted because they would be outside of the software's calibration range.

Methods S3

CellProfiler Module Description

CellProfiler 3.1.8 was used as downloaded from cellprofiler.org on a Windows 10 PC. Modules were added or removed using the software's interface, with no outside modification. The modules have been outlined here in order to be reproduced as needed.

- Images: Loads images for analysis
- Metadata: Allows extraction of metadata from image file
- Names and Types: Assigns a variable name to the currently loaded image for reference by later modules
- Groups: Allows data grouping. Not used by default
- Gaussian Filter: Applies Gaussian blur filter to the image at sigma = 4
- Enhance and Suppress Features: Used the "speckles" option with size based on object size
- Crop1 and Crop2: Images are loaded as whole pictures but must be analyzed by halves. These two modules crop the blurred image into a left and right side, which is carried forward by the software. An x value was chosen based on the average width of the image resolution.
- IdentifyPrimaryObjects1,2,3: This module identifies cells by finding a threshold, adjusting it based on an empirical factor, and then de-clumps the cells based on other empirically identified parameters. The range of expected cell sizes is input here as well.
    - Multiple identify primary object modules are used. In the first, the brightness threshold is algorithmically identified based on the entire image. After doing this, the left and right side should not have different thresholds, so they use the one calculated for the whole.
- Measure Object Intensity: This module calculates the brightness contained inside each identified cell.
- Measure Image Intensity: This module calculates various brightness measurements of the images as a whole.
- Rescale Intensity: The raw images are typically very dim, which makes visual comparison difficult. For easy analysis and quality of life, this module automatically re-scales the intensity of the raw image.
- Overlay Objects: This module produces the colorful images shown throughout this work. This allows for easy quality checking, as it shows which cells were counted on top of the brightened image.
- Save Images: This selects a name and location for saving the produced images.
- Export to Spreadsheet: This step exports .csv files that contain all the data specified. By default, it will export all data all the time, but many of the reported measurements are redundant.

Table S1. List of plasmid constructs used in this study

Construct	Purpose	Gene Cassette 1	Gene Cassette 2	Source
pLMNC95	GFP reporter	P35S::erGFP7^{INT}-Tnos		[2]Mankin and Thompson, 2001
pKL2187	Dual fluorescence reporter	2xP35S::tdTomato-Tnos	2xP35S-Ω::ZsGreen1-TpinII (+1)	This study
pKL2188	Dual fluorescence reporter	2xP35S::tdTomato-Tnos	2xP35S-Ω::ZsGreen1-TpinII	This study
pTF6005	Targeted Cas9 editing of pKL2187	PZmUbi::SpCas9-T35S	POsU6::OsPDS-gRNA1	[3]Banakar et al., 2019
pTF6005-1	Targeted Cas9 editing of pKL2187	PZmUbi::SpCas9-T35S	POsU6::OsPDS-gRNA2	This study
pTF6005-2	Targeted Cas9 editing of pKL2187	PZmUbi::SpCas9-T35S	POsU6::OsPDS-gRNA3	This study
A845B	Negative control for Cas9 targeted editing	PZmUbi::SpCas9-rbcS-E9t	POsU3::ZmGl2-gRNA2	[4]Lee et al., 2019

References:

1. Widholm, J. M. The use of fluorescein diacetate and phenosafranine for determining viability of cultured plant cells. *Biotech. Histochem.* **1972**, *47* (4), 189–194. https://doi.org/10.3109/10520297209116483.
2. Mankin, L.S. and Thompson, W.F. (2001). New green fluorescent protein genes for plant transformation: intron-containing, ER-localized, and soluble-modified. *Plant Mol. Biol. Rep.* 19:13–26
3. Banakar, R., Eggenberger, A.L., Lee, K., Wright, D., Murugan, K., Zarecor, S. *et al.* High-frequency random DNA insertions upon co-delivery of CRISPR-Cas9 ribonucleoprotein and selectable marker plasmid in rice. *Sci. Rep.* 9, 19902 (2019). https://doi.org/10.1038/s41598-019-55681-y
4. Lee, K., Zhang, Y., Kleinstiver, B. P., Guo, J. A., Aryee, M. J., Miller, J., et al. (2019). Activities and specificities of CRISPR-Cas9 and Cas12a nucleases for targeted mutagenesis in maize. *Plant Biotechnol. J.* 17, 362-372.

Table S2. Results of cell counting by hand compared to CellProfiler

**Image #1**	**Person1**	**Person2**	**Person3**	**CellProfiler**
Left side	278	182	281	324
Right side	323	193	284	380
Ratio	1.16	1.06	1.01	1.17

**Image #2**	**Person1**	**Person2**	**Person3**	**CellProfiler**
Left side	28	23	30	25
Right side	38	29	37	37
Ratio	1.36	1.26	1.23	1.48

**Image #3**	**Person1**	**Person2**	**Person3**	**CellProfiler**
Left side	335	295	368	451
Right side	191	140	180	253
Ratio	0.57	0.47	0.49	0.56

**Image #4**	**Person1**	**Person2**	**Person3**	**CellProfiler**
Left side	172	122	147	206
Right side	117	82	95	145
Ratio	0.68	0.67	0.65	0.70

**Image #5**	**Person1**	**Person2**	**Person3**	**CellProfiler**
Left side	168	125	172	154
Right side	22	22	24	24
Ratio	0.13	0.18	0.14	0.16

**Image #6**	**Person1**	**Person2**	**Person3**	**CellProfiler**
Left side	173	121	158	182
Right side	117	88	120	117
Ratio	0.68	0.73	0.76	0.64

**Image #7**	**Person1**	**Person2**	**Person3**	**CellProfiler**
Left side	153	109	159	206
Right side	175	151	182	214
Ratio	1.14	1.39	1.14	1.04

Supplemental Figures

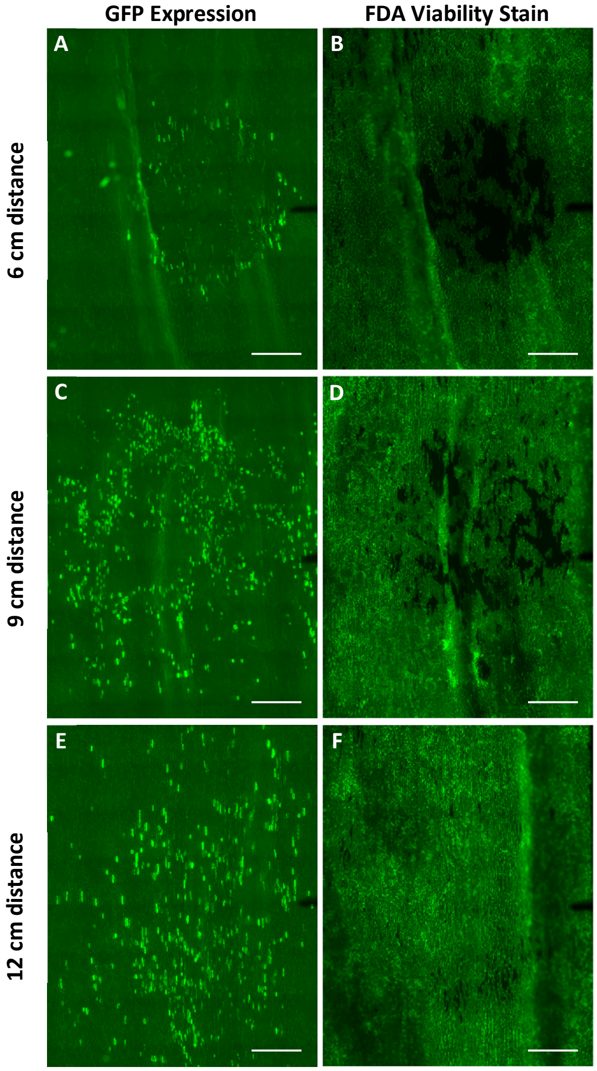

Figure S1. The effect of S-T distance on DNA delivery and cell viability when using the double-barrel device. DNA delivery measured by GFP expressing cells using S-T distance of 6 cm **(A)**, 9 cm **(C)** and 12 cm **(E)**. Cell viability assessment using fluorescein diacetate (FDA) on onion tissue when bombarded using S-T distance of 6 cm **(B)**, 9 cm **(D)** and 12 cm **(F)**. Green and dark areas after FDA staining are indicative of live and dead cells, respectively. Scale bar is 4 mm in all images.

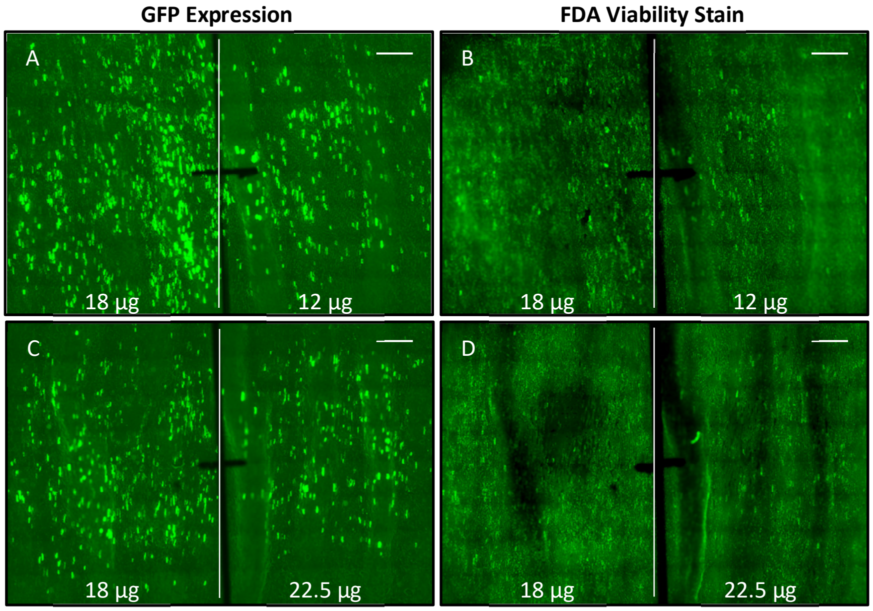

Figure S2. Side-by-side comparison of the quantity of gold particle per shot on DNA delivery and cell viability when using the double-barrel device. DNA delivery measured by GFP expressing cells in onion tissue bombarded with 18 µg/shot vs 12 µg/shot **(A)** and 18 µg/shot vs 22.5 µg/shot **(C)**. **(B, D)** Cell viability assessment using fluorescein diacetate (FDA) staining of the tissue samples in (A) and (C), respectively. Green and dark areas after FDA staining are indicative of live and dead cells, respectively. All bombardments were carried out using 650 psi rupture disks and 12 cm S-T distance. Scale bar is 4 mm in all images.

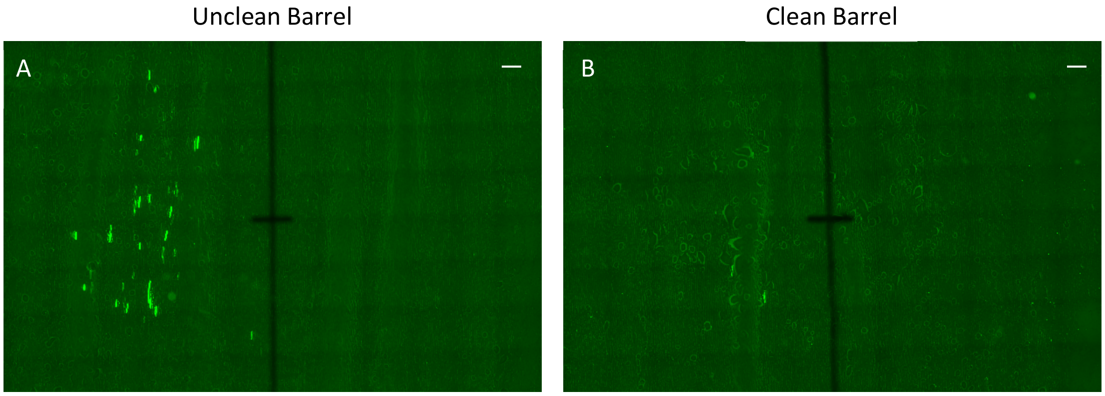

Figure S3. Effect of barrel-cleaning between shots on level of incidental fluorescence carryover. Images of blank shots (no gold or DNA on macrocarrier) performed without barrel-cleaning **(A)** and with barrel-cleaning step **(B)** after previous bombardment of DNA samples with a positive control in-frame *gfp* construct pKL2188 on the left and a CRISPR/reporter mix on the right. Scale bar: 4 mm.

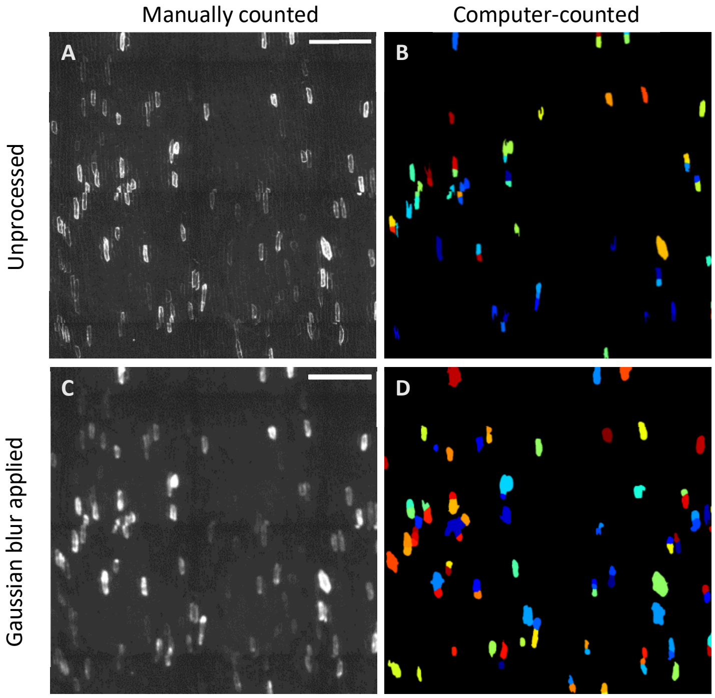

	Hand-count	CP Count (unprocessed)	CP Count (blurred)
Outcomes	74	48	79

Figure S4. Smoothing optimization. (A) A cut-out of a fluorescent image as taken by the microscope software, looking at a typical transfected onion tissue. 74 cells were found in this image when counted by hand; **(B)** The software overlay indicating which cells were counted (each color is a separate cell), and the total number of cells; **(C)** The image when filtered with a Gaussian blur using $\sigma = 4$; **(D)** The resulting counted image with the software overlay. Scale bar is 2 mm. The table shows the number of cells seen in each image, counted either by hand or by the software.

A. De-clumping modes

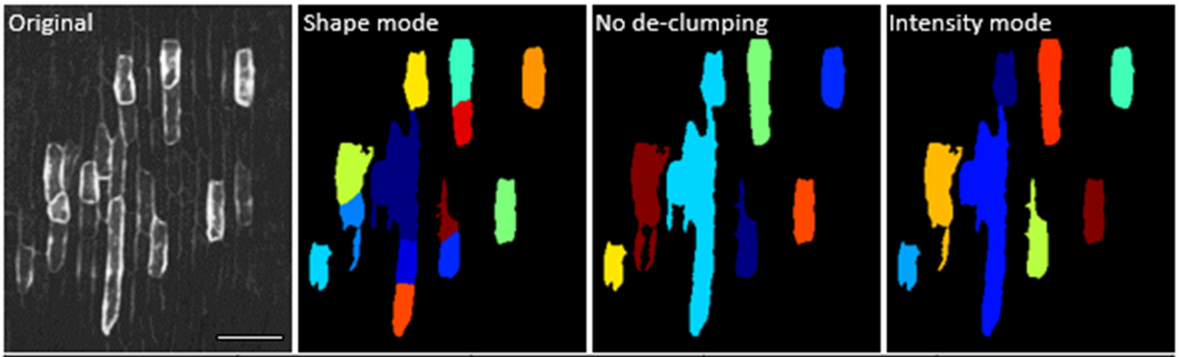

	Hand-count	No de-clumping	Shape mode	Intensity Mode
Count Outcomes	13	7	13	8

B. Minimum distances

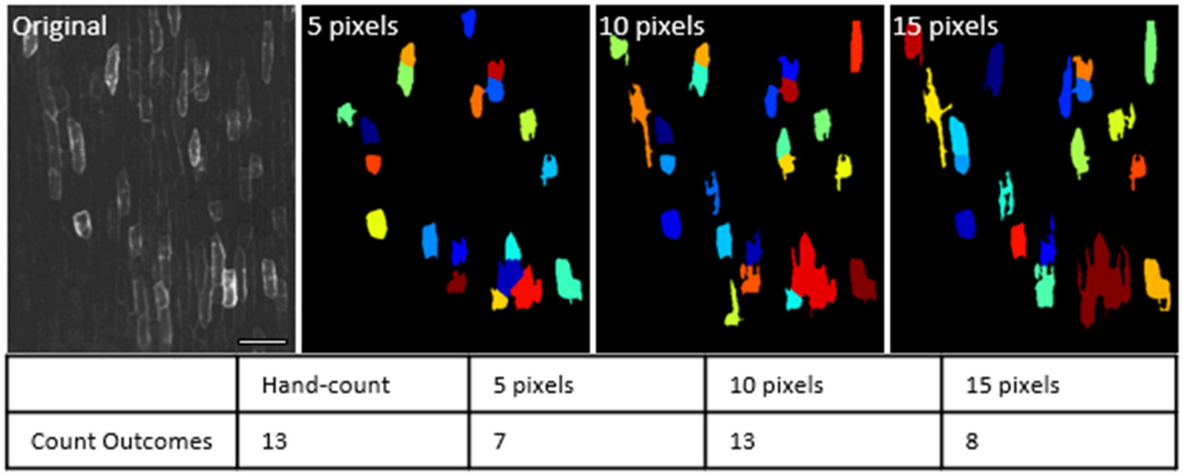

	Hand-count	5 pixels	10 pixels	15 pixels
Count Outcomes	13	7	13	8

Figure S5. De-clumping optimization. (A) An original fluorescent image and the software overlays showing the effect of different modes on distinguishing cells, and the respective number of cells counted; **(B)** A similar series of images showing the effect of the "minimum distance" setting on distinguishing cell boundaries. Scale bars are each 0.5 mm. Tables show the number of cells counted either by hand or by the algorithm with the different settings.

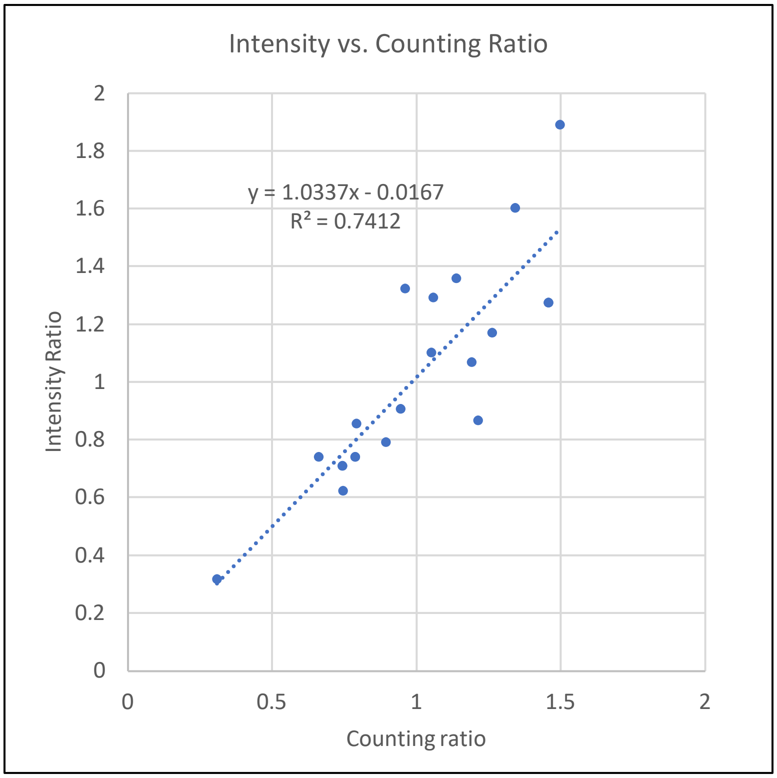

Figure S6. Comparison of performance ratio based on number of cells to brightness. The CellProfiler software can both count the number of cells and capture the brightness (integrated density) of cells in the counted area. The chart compares the performance ratio for different methods of characterizing transfection and finds them highly correlated.

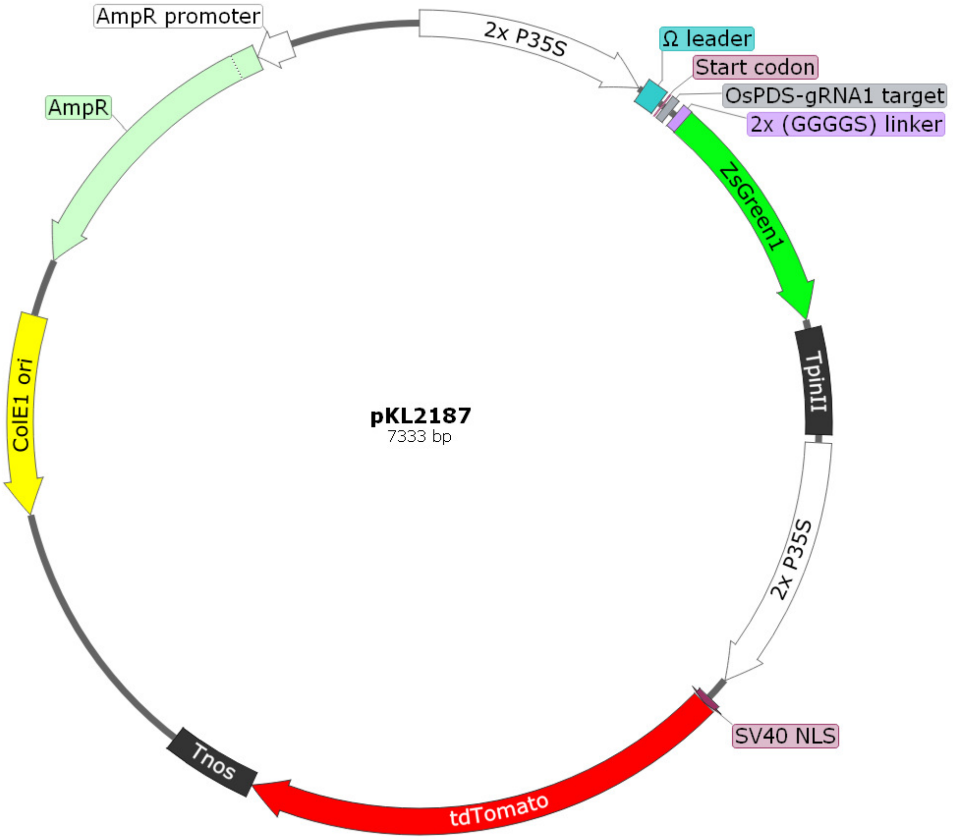

Figure S7. Map of the reporter plasmid pKL2187. The plasmid has genes for the red fluorescent protein tdTomato and the green fluorescent protein ZsGreen1. Transcription of the tdTomato gene is driven by a 2X CaMV 35S promoter (P35S) and terminated by an *Agrobacterium* nopaline synthase terminator (Tnos). The encoded tdTomato protein has an SV40 nuclear localization signal at the N-terminus. Transcription of the ZsGreen1 gene is driven by a 2X P35S promoter and terminated by a potato protease inhibitor II terminator (TpinII). The translation start codon is preceded by a TMV Ω translational enhancer and is immediately followed by the target sequence of OsPDS-gRNA1 expressed from pTF6005. The open reading frame for the flexible peptide linker 2X (GGGGS) and ZsGreen1 is out-of-frame with the start codon and is not translated, however indel mutations at the gRNA target site can bring the ZsGreen1 gene in-frame and restore green fluorescence. ColE1 ori, high copy number origin of replication for *E. coli*. Amp^R, ampicillin resistance gene.

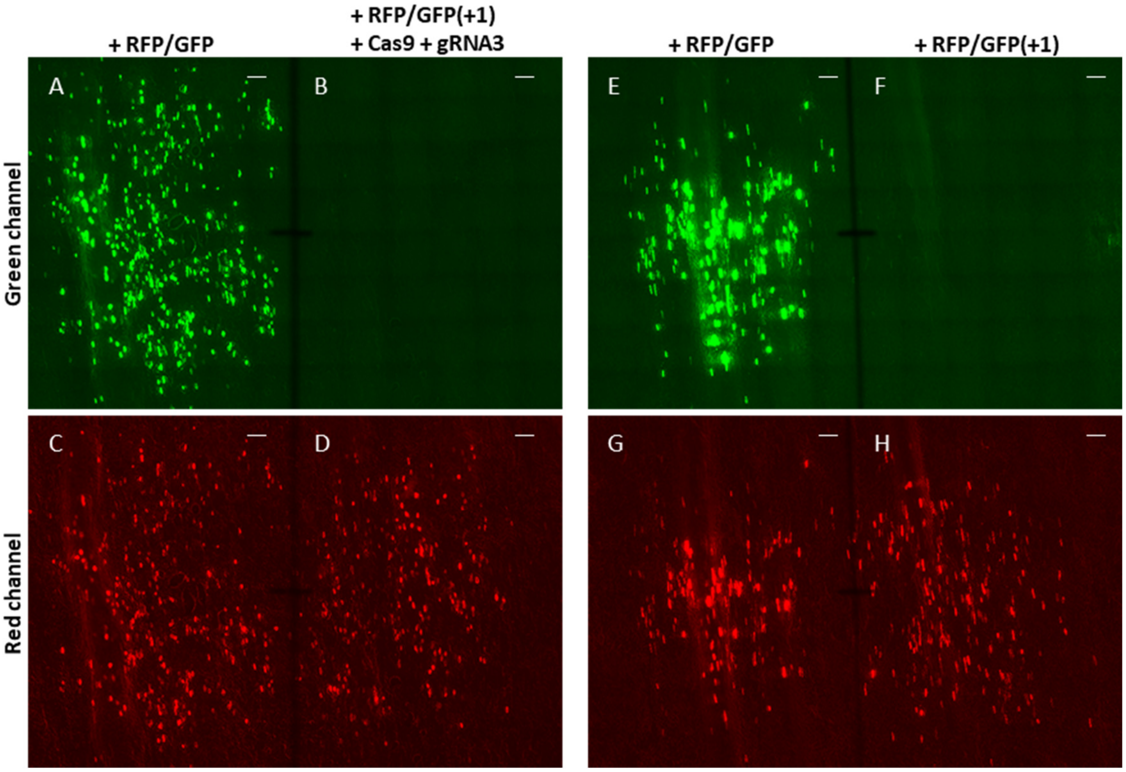

Figure S8. Cas9 editing with gRNA3. (A, C, E, G) Samples were bombarded with plasmid pKL2188 (+RFP/GFP) that had in-frame *gfp* and *rfp* genes. **(B, D)** Co-bombardment of reporter construct pKL2187 (+RFP/GFP+1) and the pTF6005-2 plasmid with Cas9 and gRNA3; **(F, H)** Co-bombardment of pKL2187 with a negative control Cas9 construct. Scale bar is 4 mm.

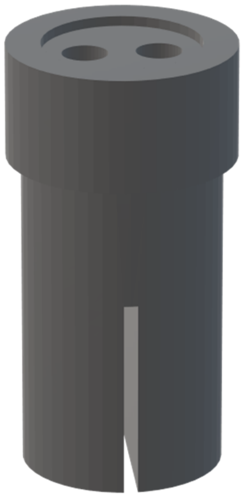

Figure S9. Double-barrel 3D model. This image is a 3D render of the STL file included as Supplemental File S1. It was developed based on a device obtained from the original developer, Dr. Brett Tyler, modified to use a single ¾ inch stopping screen instead of two 5/8 inch stopping screen. Reproduced here with permission. It is fully compatible with the standard Bio-Rad PDS-1000 gene gun.

Supplemental File S1: 3D-printable double-barrel file (.stl)
